# Temperate forest heterogeneity decreases local and landscape‐scale spider diversity through habitat filtering despite increasing species turnover

**DOI:** 10.1111/1365-2656.70297

**Published:** 2026-06-26

**Authors:** Jean‐Léonard Stör, Julia Rothacher, Marc W. Cadotte, Anne Chao, Maike Huszarik, Michael Junginger, Lisa Köstler‐Albert, Oliver Mitesser, Akira S. Mori, Clara Wild, Jörg Müller

**Affiliations:** ^1^ Department of Animal Ecology and Tropical Biology, Biocenter University of Würzburg Würzburg Germany; ^2^ Chair of Conservation Biology and Forest Ecology, Biocenter Julius‐Maximilians‐Universität Würzburg Rauhenebrach Germany; ^3^ University of Toronto Toronto Ontario Canada; ^4^ Institute of Statistics National Tsing Hua University Hsin‐Chu Taiwan; ^5^ Animal Population Ecology, Bayreuth Center for Ecology and Environmental Research (BayCEER) University Bayreuth Bayreuth Germany; ^6^ Research Center for Advanced Science and Technology The University of Tokyo Tokyo Japan; ^7^ Bavarian Forest National Park Grafenau Germany

**Keywords:** forest conservation, functional diversity, habitat heterogeneity, landscape ecology, structural complexity

## Abstract

1. Spiders are important arthropod predators in temperate forests. Their diversity depends on structurally heterogeneous habitats offering diverse microhabitats. Yet, modern silviculture has homogenized temperate forest structure at local and landscape scales. The consequences of this homogenization for landscape‐level spider diversity, however, remain largely unknown.
2. We sampled spiders using pitfall traps across 234 patches in a large‐scale, replicated field experiment at 11 forest sites across Germany. At each site, one treatment district was experimentally heterogenized through canopy gap creation, thinning and deadwood enrichment, and a second homogeneous district remained untreated as a control.
3. We applied a novel meta‐analytic framework to compare *α*‐, *β*‐ and *γ*‐diversity of spiders between treatment and control districts, standardized for sample coverage along Hill numbers giving increasing weight to abundance and included taxonomic, functional and phylogenetic diversity facets. We also investigated spider community assembly in response to deadwood enrichment, canopy openness and heterogeneous forest structure.
4. Based on 18,540 spider individuals from 206 species, treatment districts exhibited significantly lower *γ*‐ and *α*‐diversity across all diversity facets and Hill numbers, particularly when focusing on rare species (*q* = 0). In contrast, *β*‐diversity increased in treatment districts for phylogenetic and functional diversity across Hill numbers (*q* = 0, 1, 2). The simultaneous decrease in *α*‐ and *γ*‐diversity despite higher *β*‐diversity renders the increase in compositional turnover insufficient to compensate for local diversity losses. Although spiders were more abundant in treatment patches, habitat filtering, rather than niche competition, shaped the community.
5. Our findings corroborate previous results of high spider abundances but lower taxonomic and functional diversity in canopy gaps due to strong habitat filtering effects. However, we demonstrate for the first time that this lower *α*‐diversity is linked to a lower *γ*‐diversity despite increases in *β*‐diversity. Homogenous forests support higher *γ*‐diversity through greater three‐dimensional canopy habitat availability. Yet, failure to account for species frequencies using Hill numbers and coverage standardization may result in a substantial underestimation of arboreal spider diversity in pitfall traps. Nonetheless, higher abundances in heterogeneous forests point towards increased prey availability and predator pressure.

## Graphical abstract

In a large‐scale German forest experiment, habitat heterogeneity increased spider turnover but reduced local alpha diversity through habitat filtering. Together, these effects lowered landscape‐scale spider diversity. As such, the study shows whether environmental heterogeneity increases or decreases biodiversity depends on the balance between habitat filtering and niche overlap within meta‐communities.

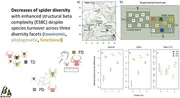

## 1 INTRODUCTION

Silviculture has largely homogenized forests across Central Europe. Since the mid‐20th century, a focus on closed‐canopy forestry has resulted in forests being maintained at an intermediate successional stage for timber production over large areas. These forests are regularly characterized by closed canopies and low amounts of deadwood (Müller et al., 2023). Yet diverse early‐ and late‐successional communities across multiple kingdoms of life, including arthropods, depend on open canopies and deadwood structures (Hilmers et al., 2018; Perry et al., 2018). In turn, the loss of structural and microhabitat heterogeneity in forests has led to the homogenization of communities and puts many species at risk (Hilmers et al., 2018; Seibold et al., 2015).

Forest management can promote heterogeneity to enhance biodiversity. At the landscape scale, homogeneous stands of different succession stages can create a heterogeneous mosaic with strong contrasts in canopy structure and microclimatic conditions, fostering distinct communities (*β*‐diversity) that may collectively enhance *γ*‐diversity (Heidrich et al., 2020; Schall et al., 2018; Uhl et al., 2022). Alternatively, structural heterogeneity can be created at smaller scales via interventions emulating natural disturbances. By increasing local light and deadwood through artificial gaps and old‐growth features (Keeton, 2006; Müller et al., 2023), these interventions provide diverse microhabitats that promote local (*α*) diversity (MacArthur & Wilson, 1967; Rothacher et al., 2023; Srivastava & Lawton, 1998). This might scale up to a higher diversity at the landscape level (Massó Estaje et al., 2026; Mitesser et al., 2026). Thus, increases in both local *α*‐ and between‐patch *β*‐diversity can drive *γ*‐diversity. Yet, recent global meta‐analyses using correlative data have shown that while more heterogeneous and well‐connected habitats exhibit higher *γ*‐diversity, these gains were primarily linked to increased *α*‐diversity, whereas *β*‐diversity responses remain unpredictable across taxa and spatial scales (Gonçalves‐Souza et al., 2025; Keck et al., 2025). This highlights the need for experimental studies that systematically integrate *α*‐ and *β*‐diversity to better understand their contributions to *γ*‐diversity and thereby the scale dependence of biodiversity responses.

Spiders (Araneae) are diverse predators and key pest control agents in terrestrial ecosystems, including temperate forests (Michalko et al., 2019). At the *α*‐level, spider abundance and observed species number often decrease with forest structural heterogeneity (Heidrich et al., 2020; Plath et al., 2024) and increase with canopy openness (Müller et al., 2022; Vierling et al., 2011). Large, cursorial hunters like wolf spiders (Lycosidae) may disproportionately benefit from open forest habitats where warmer temperatures and fewer obstacles facilitate hunting. Thus, early successional forest habitats and intensively managed forests (Plath et al., 2021) can exhibit more diverse, yet fundamentally different spider assemblages than old‐growth stands (García‐Tejero et al., 2018; Plath et al., 2024). Although higher *α*‐diversity and distinctly different community composition (*β*‐diversity) are found in heterogeneous forest environments, forest spider *γ*‐diversity remains poorly understood due to a lack of well‐replicated landscape experiments. In turn, it is unclear whether spider *γ*‐diversity of heterogeneous forests is shaped predominantly by *α*‐ or *β*‐responses.

Integrating taxonomic (TD), functional (FD) and phylogenetic (PD) diversity provides a multifaceted understanding of forest spider responses. Functional and evolutionary dimensions of diversity are tightly coupled, since many spider traits are phylogenetically conserved, and jointly influence community assembly (Cadotte et al., 2013; Michalko et al., 2016). However, as traits and phylogeny might not strongly correspond because of trait convergence or rapid divergence (Cadotte et al., 2017, 2019), community assembly relies on both. Nonetheless, research has largely focused on abundance and observed species number whereas integrative assessments of functional and phylogenetic diversity and unbiased diversity estimates remain limited (but see Heidrich et al., 2020; Müller et al., 2022), constraining inference on how forest heterogeneity shapes coexistence beyond taxonomic metrics. Deadwood heterogeneity should enhance functional and phylogenetic *α*‐diversity by increasing niche availability and promoting phylogenetically distant taxa (Buddle & Shorthouse, 2008), as 80% of forest spiders use deadwood at least facultatively (Graf et al., 2022). Furthermore, saproxylic spider community composition is strongly shaped by deadwood microclimates (Müller et al., 2018). In turn, canopy‐driven microclimatic filtering may elevate *β*‐diversity by favouring closely related, functionally similar species across sun–shade gradients, such as thin‐legged wolf spiders (*Pardosa* spp., Michalko et al., 2016). The integration of these additional dimensions of diversity clarifies species assembly processes and facilitates the identification of functional redundancy.

In this study, we made use of one of the largest temperate forest landscape manipulation experiments in Europe (Müller et al., 2023) to assess how forest heterogeneity impacts three facets of spider diversity (i.e. TD, FD and PD) across *α*‐, *β*‐ and *γ*‐scales. In a paired design, silvicultural interventions heterogenized formerly homogeneous broad‐leaved forests by increasing canopy openness and deadwood variety, both important features of natural old‐growth stands (Bauhus et al., 2009; Donato et al., 2012) and important in forming spider habitats (Graf et al., 2022; Müller et al., 2018, 2022). Specifically, we hypothesized that: (i) structural heterogeneity enhances spider diversity at both the patch scale (*α*‐diversity) and the landscape scale (*γ*‐diversity) compared with structurally homogeneous control forests; and (ii) increased structural heterogeneity promotes the assembly of differing spider assemblages across patches, resulting in higher between‐patch *β*‐diversity in heterogeneous forests.

## 2 MATERIALS AND METHODS

### 2.1 Study area and experimental design

**FIGURE 1:**
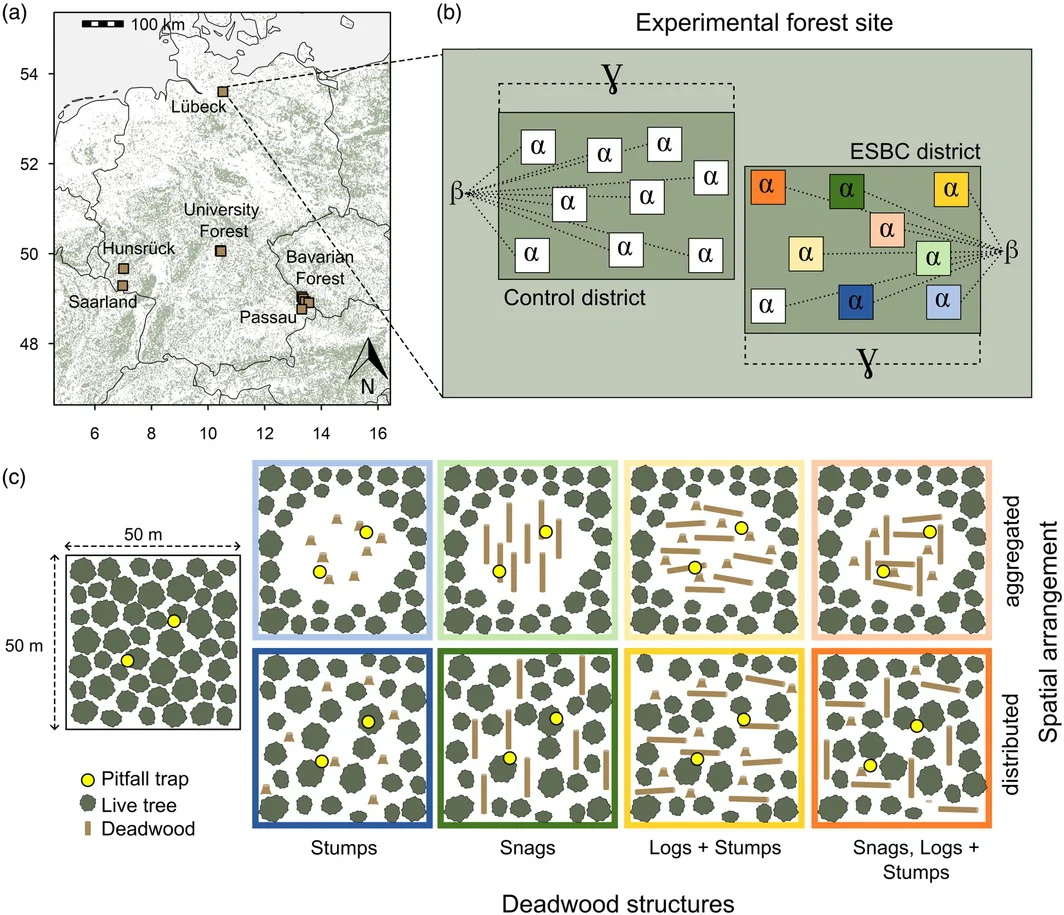
Experimental design of the ΒETA‐FOR research unit. (a) Location of 11 experimental sites across six German regions (brown rectangles indicating sites; forest cover in green). Three sites are located in the University Forest and four sites in the Bavarian Forest. (b) Schematic representation of an experimental forest site, featuring a structurally heterogeneous treatment (ESBC) district and a homogeneous control district. (c) Overview of the nine treatment types realized in all treatment districts. The positions of pitfall traps in each patch (north‐east and south‐west) are indicated by yellow dots. Note, six additional treatment types were applied exclusively in the University Forest (see main text and Figure S1).

This study is part of the collaborative research unit ΒETA‐FOR and was conducted at 11 experimental forest sites across Germany, all dominated by European beech (*Fagus sylvatica*) with smaller, site‐specific proportions (1%–30% of total basal area) of other broad‐leaved and coniferous species. The forest sites are located in the management zone of the Bavarian Forest National Park (4 sites: B04, B05, B06 and B07), the University Forest of the Julius‐Maximilians‐Universität Würzburg (3 sites: U01, U02 and U03), the management zone of the Hunsrück‐Hochwald National Park (1 site: H09) and the administrative districts of Passau (1 site: P08), Saarland (1 site: S10) and Lübeck (1 site: L11) spanning a broad elevational gradient from 38 m (Lübeck) to 1143 m a.s.l. (Bavarian Forest, Figure 1). Consequently, the experimental design captures a wide range of macroclimatic conditions, soil types and tree species compositions representative of Central European beech forest types, enabling broadly applicable insights into how forest structure influences biodiversity in these ecosystems.

Two experimental districts of about 20 ha were established in the forest interior at each site, corresponding to the size of an equilibrium landscape in temperate forests based on forest gap dynamic models (Bugmann et al., 1996). Within each district, a set of experimental patches was established. Sites in the Bavarian Forest, Passau, Saarland, Lübeck and Hunsrück each comprised 18 patches of 50 m × 50 m in size (nine patches per district), while sites in the University Forest contained 30 patches (15 patches per district). In total, this design resulted in 234 experimental patches across Germany.

We experimentally enhanced structural beta complexity (ESBC) in one district per site (treatment district) through targeted silvicultural interventions, whereas the second district was left untreated as a homogenous control. The silvicultural interventions were implemented in the winters of 2015/2016 (Bavarian Forest, Passau), 2016/2017 (Saarland, Lübeck, Hunsrück) and 2018/2019 (University Forest) (Rothacher, Seidl, et al., 2025). Interventions were performed either spatially aggregated by creating canopy gaps (~30 m in diameter) in the patch center of about 700 m^2^, approximating the typical patch size in beech forests predicted by theory (Bugmann et al., 1996) or distributed throughout the patch thereby maintaining a closed canopy. In both spatial settings, interventions targeted the manipulation of 30% of the total basal area timber stock per patch and created different deadwood structures (i.e. stumps, snags, logs and a combination thereof). These manipulations resulted in nine distinct treatment patch types per site (see Figure 1). In the University Forest, six additional treatments were applied, including aggregated and distributed stump extraction, habitat tree creation and crown retention (see Figure S1 for details).

The control and treated districts of each site had the same number of patches and were located in close proximity, sharing a similar elevation and tree species composition. Since no interventions were applied, control patches closely resembled structurally homogeneous production forests, as commonly found across Central Europe. They were characterized by mature trees of similar age, in the early to mid‐optimum stage of stand development, a closed canopy and a limited amount of deadwood. This large‐scale experimental approach enabled assessing local *α*‐diversity within each of the 234 patches, between‐patch *β*‐diversity, and overall *γ*‐diversity of each treatment and control district per forest site (Figure 1).

### 2.2 Spider sampling

Spiders were sampled using two pitfall traps per patch. The pitfall traps were installed in the central area of each patch (25 × 25 m) along a southeast and northwest axis to represent the patch conditions (Figure 1). Although primarily designed for ground‐dwelling arthropods, pitfall traps also effectively sample vegetation‐dwelling and arboreal spider species (Figures S15 and S16) that descend from trees or shrubs during the mating season (Müller et al., 2022). Each pitfall trap consisted of a 400 mL plastic cup buried at ground level. We covered each trap with a transparent plastic roof (15 × 15 cm, approximately 5 cm above the ground), to protect the trap from rain or leaves. We used a solution of 15% sodium chloride (NaCl) as the trapping fluid and added a drop of odourless detergent to break the surface tension, which ensured rapid killing of the sampled arthropods. The traps were active for three consecutive months of high adult spider activity (i.e. May, June and July) in 1 year and emptied once per month. Patches in the Bavarian Forest, University Forest and Passau were sampled in 2022, whereas patches in Saarland, Lübeck, Hunsrück were sampled in 2023 with permits obtained from the respective regional authorities (Regierung von Niederbayern; Regierung von Unterfranken; Ministerium für Umwelt, Klima, Mobilität, Agrar und Verbraucherschutz Saarland; Landesamt für Umwelt Schleswig‐Holstein). The collected samples were sorted and spiders from each sample were identified following Roberts (1985) by the first author. The identification of difficult specimen was confirmed by a leading spider expert (Dr. Ingmar Weiß, Grafenau, Germany). Species‐level identification was achieved for ~96% of the individuals, resulting in the analysis of 18,540 spiders across 206 species, which represents ~20% of Germany's spider fauna (Blick et al., 2016).

### 2.3 Data preparation

**FIGURE 2:**
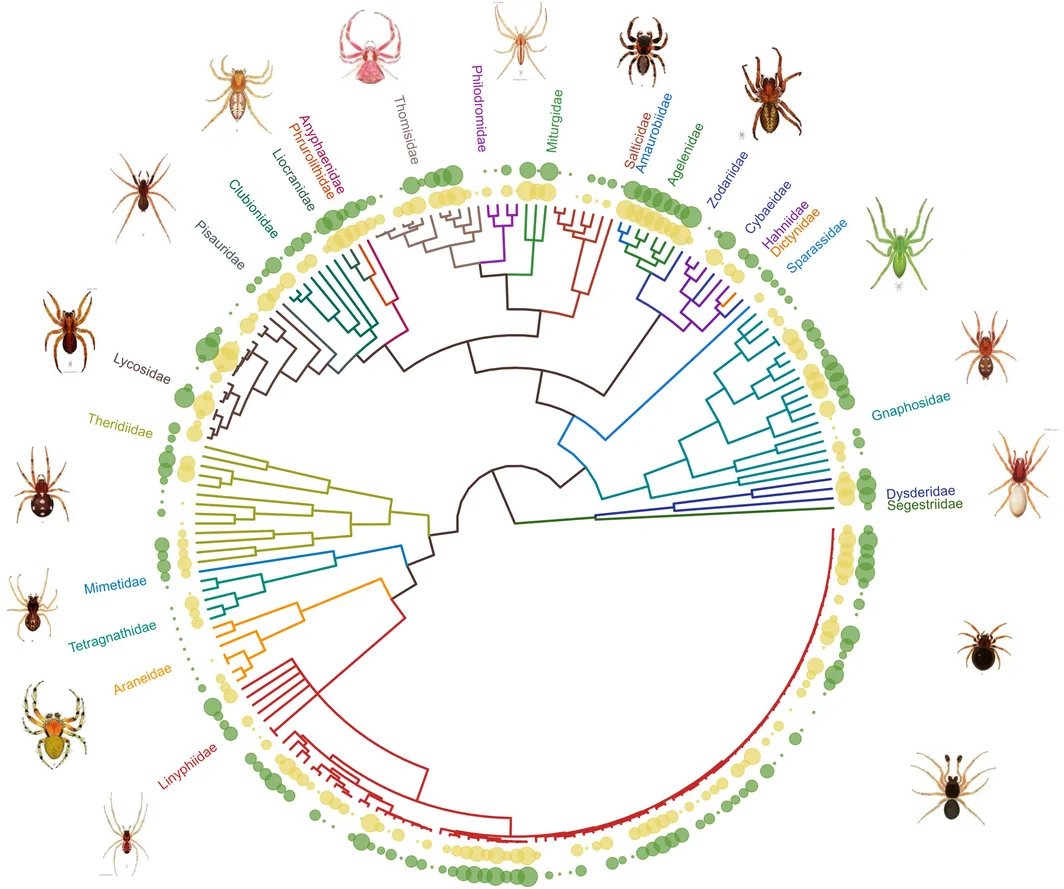
Phylogenetic tree of 206 spider species from the forest manipulation experiment. Tips represent individual species, with branch colours indicating family associations. Dot size denotes total abundance within ESBC (yellow) and control (green) districts. Spider family illustrations adapted from Roberts (1985).

Data preparation and statistical analyses were performed in R 4.5.2. (R‐Core‐Team, 2025). To eliminate potential biases from juvenile‐heavy taxa (22.7% of all sampled individuals were juveniles which cannot be reliably identified to species level), we restricted the dataset to adult spiders by aggregating the data (males and females) on patch level. For phylogenetic diversity analyses, we constructed a phylogenetic tree encompassing all recorded species (Figure 2; see Supporting Information for details).

To expand the trait dataset from Müller et al. (2022), we integrated 63 additional spider species, incorporating ecological traits (hunting strategy, vertical stratum, ballooning ability and mean body size) and morphological measurements (Table 1). For new species, ecological data were sourced from literature, while morphological traits were measured directly. To ensure morphological values were independent of nutritional or developmental history, we standardized all measurements to body length by extracting residuals from linear regressions of log_e_(trait) ~ log_e_(body length). Functional distances between species were then calculated using Gower distance (Cadotte et al., 2013; Müller et al., 2022), which accommodates both continuous and categorical variables through the dissimilarity coefficient of Gower (1971). To ensure equal weighting across the dataset, multi‐category traits (e.g. vertical stratum) were down‐weighted accordingly (see R‐code for details).

**TABLE 1:** Traits characterizing the behaviour of spider species in response to local environments.

Trait	Ecology	Measurement	Source
Stratum	Spiders use different strata in a forest from ground to canopy layer	1/0 coding of 5 forest strata: epigeic, herb layer, shrub layer, tree trunk, canopy layer; multiple strata per species possible	Müller et al. (2022) and additional information from literature
Web‐type	Spiders use different types of web for foraging	Five categories: no web, web with tangles of stopping threads, horizontal web bottom, horizontal web top, vertical web	Müller et al. (2022) and additional information from literature
Ballooning	Some spiders use passive ballooning for long‐distance dispersal	1/0 coding of the ballooning high and ballooning low probability	Birkhofer et al. (2015) and additional information from literature
Body size	Body size decreases from warm and dry to cool and moist conditions, body size increases with habitat complexity	Continuous values from literature	Entling et al. (2010) and additional information from literature
Opisthosoma and prosoma breadth	Arthropods are narrower in structurally more complex habitats	Residuals of a linear model loge(*y*) ~ loge(*x*): Opisthosoma and prosoma breadth (mm) ~ body length (mm) measured	Gibb et al. (2015) and additional information from literature
Leg length	Might decrease in structurally simple habitats	Residuals of tibia length of first leg as before	Gibb et al. (2015) and additional information from literature
Fang length	Fast prey in less complex and warmer environment evokes long fang length	Residuals of chelicera length as before	Gibb et al. (2015) and additional information from literature

### 2.4 Statistical analysis

To evaluate differences in taxonomic (TD), phylogenetic (PD) and functional diversity (FD) between ESBC and control districts, we employed a comparative meta‐analysis framework using the *iNEXT.3D* approach (Chao et al., 2021) that integrates sample coverage (an objective and rigorous measure of sample completeness) and Hill numbers (Hill, 1973).

Because diversity data are regularly incomplete and arthropod sample coverage can vary systematically with canopy treatment (Rothacher, Seidl, et al., 2025) or along succession gradients (Kortmann et al., 2025), standardizing to a common coverage level within a unified analytical framework is necessary to guarantee meaningful diversity comparisons (Chao & Jost, 2012). Without such correction, diversity comparisons across sites may be biased and apparent treatment effects may simply reflect coverage differences. The incorporation of Hill numbers allows a focus on rare (*q* = 0), common (*q* = 1) and dominant (*q* = 2) species, as well as lineages and functional units in the dataset. In our case, rarity partly reflects the sampling method, with rare species largely representing vegetation‐ and arboreal‐dwelling taxa, whereas common and dominant species are mainly ground‐dwelling. We first calculated meta‐analyses for TD, FD and PD across Hill numbers on the district level (*γ*‐diversity, landscape level) with 99 bootstrapping replications using the standardized coverage value of 0.76 calculated by the *iNEXTmeta* function for sample coverage standardization (Chao, 2025; Massó Estaje et al., 2026; Mitesser et al., 2026). Then, we decomposed *γ*‐diversity (landscape level) into *α*‐ (patch level) and multiplicative *β*‐components using *iNEXT.beta3D* (Chao et al., 2023). We performed this analysis on the dataset containing all 234 patches, but conducted a robustness analysis using only 198 patches, after excluding the six additional treatments realized only in the University Forest (Figure S13). Detailed results (Figures S2–S10), and further information on the meta‐analysis framework are provided in the Supporting Information.

To examine treatment effects on *α*‐diversity for each order of q within the ESBC district in more detail, we parameterized generalized additive models using the *gam* function in *mgcv* (Wood, 2017), with TDs (family = negative binomial) as the response variables and treatment as the explanatory variable (Figure S14). This allowed us to include a correlated patch‐specific intercept (geographical position of the patch) to account for the spatial arrangement within and between regions (Rothacher, Seidl, et al., 2025; Uhler et al., 2021).

To assess treatment effects on assembly processes such as functional dissimilarity at patch and district scales, we calculated standardized effect sizes of mean pairwise distances (SES.MPD). Because trait datasets often lack complete information, we followed Cadotte et al. (2013), who integrated functional and phylogenetic dissimilarity. Functional distances are combined with phylogenetic distances, which act as surrogates for unmeasured traits, via the weighting parameter *a* (0 = functional only, 1 = phylogenetic only, intermediate = both), implementing this with *funct.phylo.dist* in the *pez* package (Pearse et al., 2015). Standardized effect sizes of mean functional‐phylogenetic diversity (SES.MFPD) was then calculated from these distance matrices as the mean pairwise distance between co‐occurring species for each patch, weighted by abundance. Null models (999 randomizations of tip labels) controlled for species numbers (Cadotte & Davies, 2016; Webb et al., 2002), and SES values were obtained with the *ses.mpd* function in *picante* (Kembel et al., 2010). Values above 1.96 indicate limited niche overlap (competition for habitat, food resources, etc.), while values below −1.96 indicate habitat filtering (environment selects against certain species, Cadotte et al., 2013; Müller et al., 2022). To assess the role of deadwood and canopy openness on spider community assembly, we grouped the deadwood treatments into deadwood‐enriched (snags, logs, snags + logs, habitat trees and crown deadwood) or non‐enriched (stumps, stump removal and control). Treatment effects were analysed using generalized additive models (GAMMs; gam function in *mgcv* Wood, 2017), with predictors including treatment (ESBC vs. control), canopy (open vs. closed) and deadwood enrichment (enriched vs. non‐enriched), and a correlated patch‐specific intercept (patch coordinates) to account for the spatial arrangement within and between regions (Rothacher, Seidl, et al., 2025). Models were fitted separately for each *a*‐value.

Model *R* ^2^ values were extracted to assess explanatory power along the functional‐phylogenetic gradient. Explanatory power peaked at an *a‐*value corresponding to 10% phylogenetic contribution (see Figure S12), indicating that functional traits captured most variation, with phylogeny providing only modest improvement. This approach indicates functional similarity by low *a*‐values, as functional distances produce the highest explanatory values in the model. The *a*‐value yielding maximum *R* ^2^ was then used to calculate SES MFPD. The latter, together with the abundance of adult spiders and the number of observed adult spider species per patch, were analysed using generalized additive models, using the *gam* function from *mgcv‐*package (Wood, 2017). Models included treatment (ESBC vs. control), canopy (open vs. closed) and deadwood (enriched vs. non‐enriched) as explanatory variables, with geographical position of the patch included as a correlated patch‐specific intercept to account for the spatial arrangement within and between regions.

## 3 RESULTS

**FIGURE 3:**
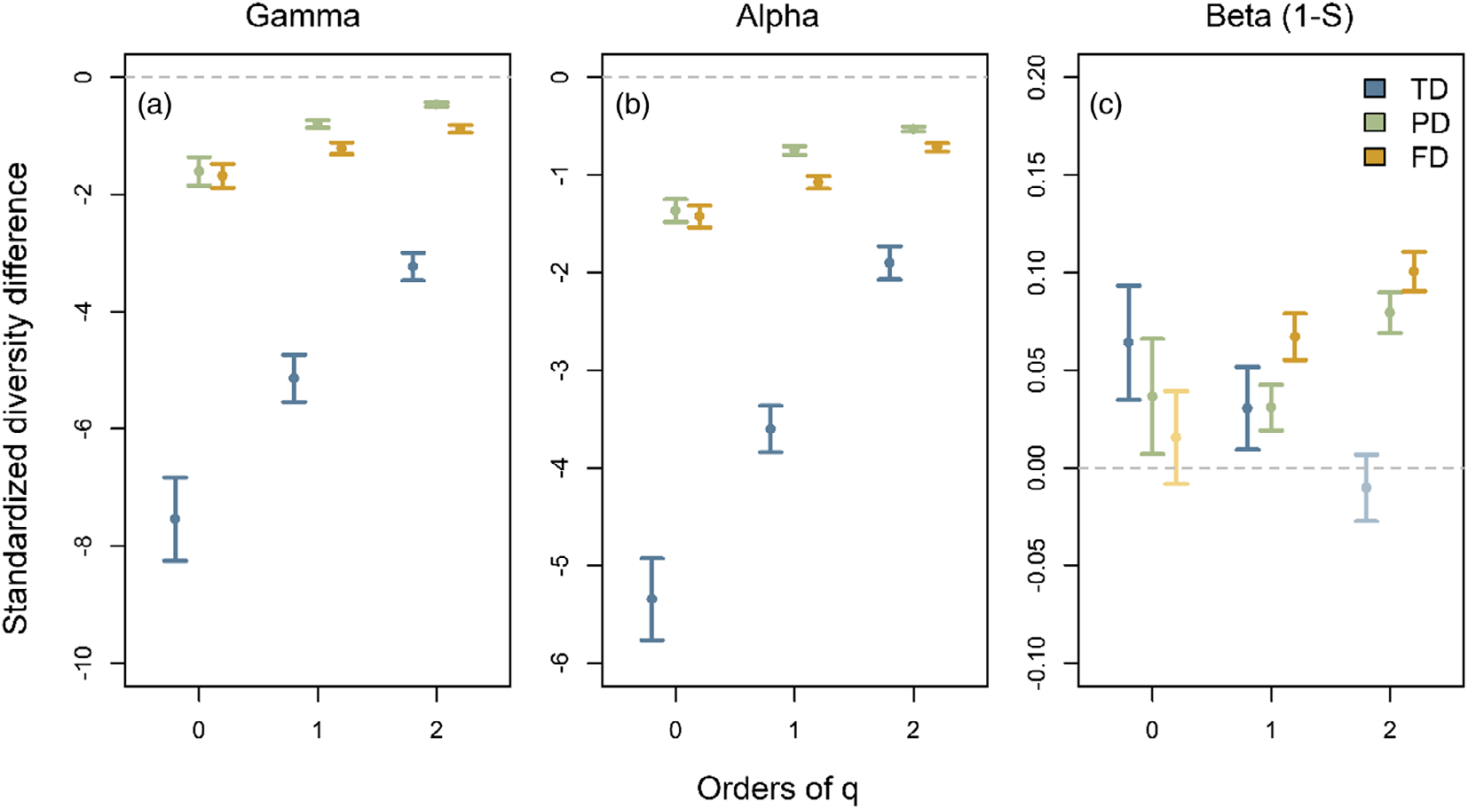
Meta‐analysis of the spider diversity response across 234 patches located in either structurally homogenous or heterogeneous districts within 11 experimental forest sites at a fixed coverage level of 0.76. Results are shown for landscape‐scale *γ*‐diversity (a), patch‐scale *α*‐diversity (b) and multiplicative (1 − S) *β*‐diversity (c). Points represent predicted means; error bars denote 95% confidence intervals. Standardized diversity differences are calculated between each pair of control and treatment districts in directly comparable species‐equivalent units for *γ*‐ and *α*‐diversity, and for taxonomic (TD, blue), phylogenetic (PD, green) and functional diversity (FD, yellow) across three diversity orders (*q* = 0, 1, 2). Positive values indicate increased diversity from control to structurally enhanced districts, negative values indicate a diversity decrease. Significant effects are shown in dark shades, non‐significant results (confidence intervals include zero) in light shades of blue, green and yellow.

Across Hill numbers, taxonomic, functional and phylogenetic *γ*‐ and *α*‐diversity were significantly lower in heterogeneous treatment districts compared with homogeneous control districts (Figure 3a,b; Figures.S2–S4 and S8–S10). This effect was strongest for taxonomic diversity, with decreases of eight effective species for *γ*‐diversity and five for *α*‐diversity. The reduction was most pronounced for rare (*q* = 0) compared with common (*q* = 1) and dominant (*q* = 2) species, lineages and functional units. Difference in taxonomic *β*‐diversity was higher for rare species (*q* = 0) but decreased with diversity order (Figure 3c; Figures S5–S7). In contrast, phylogenetic and functional *β*‐diversity differences showed an increase with diversity order and highest values for dominant functional species or phylogenetic lineages (*q* = 2, Figure 3c, Figures S5–S7). Robustness analyses restricted to the nine treatment types present at all sites supported these results (Figure S13). Nevertheless, spider *α*‐diversity remained largely unaffected by treatment type (Figure S14).

A detailed analysis of the patch‐level assembly processes revealed that spider abundance was locally higher in open‐canopy patches and at the landscape scale under ESBC treatments (Table 2). The number of observed spider species was not significantly affected by canopy condition, ESBC treatment, nor deadwood enrichment (Table 2). Standardized effect sizes of mean pairwise functional‐phylogenetic distances (SES MFPD) were strongly negative in open‐canopy patches and ESBC (Table 2), indicating environmental clustering of spider communities.

**TABLE 2:** *T*‐values from generalized additive models testing the effects of treatment (ESBC vs. Control), canopy (open vs. closed) and deadwood enrichment on spider abundance, observed species number and standardized effect size of the mean pairwise functional‐phylogenetic distance (SES MFPD, a‐value 10%).

Predictors	Abundance	Species number	SES MFPD
Family	Neg. binomial	Neg. binomial	Gaussian
ESBC versus Control District	**2.81** **	−0.61	**−3.88** ***
Open versus Closed Patch	**2.10** *	−0.41	**−2.70** **
Deadwood versus no deadwood	−0.72	−0.72	0.33

*Note*: Significant results are shown in bold.

* *p* < 0.05.

** *p* < 0.01.

*** *p* < 0.001.

## 4 DISCUSSION

Contrary to our first hypothesis, structural heterogenization reduced spider *α*‐ and *γ*‐diversity compared with control forests, although *β*‐diversity partly increased in line with our second hypothesis. Spider abundance was higher in heterogeneous stands and open canopies, but these assemblages were composed of functionally more similar species.

### 4.1 Habitat filtering and sample coverage standardization explain diversity loss in heterogeneous landscapes

Our experiment showed that structurally enhanced (ESBC) forests harboured consistently lower spider diversity than homogeneous controls, with *γ*‐diversity decreasing by up to eight effective species linked to reductions in patch‐scale *α*‐diversity. Open forest structures provide an increased amount of understorey vegetation (Bradler et al., 2025), whilst complex three‐dimensional structures of tree canopies, an important habitat for temperate forest spiders (Niedobová et al., 2025), are reduced. In line with island biogeography theory (MacArthur & Wilson, 1967), this reduction in habitat area may lower resource availability (Rothacher, Mitesser, et al., 2025) and, consequently, individual abundance and diversity, consistent with the more‐individuals hypothesis (Srivastava & Lawton, 1998). In addition, canopy gaps reduce structural complexity and thus the niche diversity available to canopy‐dwelling spiders (MacArthur & MacArthur, 1961; Wildermuth et al., 2023).

Nevertheless, our small‐scale open‐canopy patches offered valuable microhabitats typical of early‐ and mid‐successional stages, resulting in distinct species assemblages as reflected by the partly higher spider *β*‐diversity in heterogeneous forests and high spider abundances associated with gaps. Gaps favoured cursorial hunters associated with heterogeneous ground strata, like *Pachygnatha* spp. (Tetragnathidae) or numerous wolf spiders (Lycosidae, see Figure 2). Thus, ESBC patches were colonized by many species which ecologically complemented and, in some cases, even replaced closed‐forest species (Figure 2). Similar results were observed in Müller et al. (2022), where canopy gaps also hosted a functionally different set of spiders.

Unlike Tsang et al. (2025), who found *γ*‐diversity to be largely maintained despite *α*‐diversity losses, the higher *β*‐diversity in our ESBC districts failed to offset this *α*‐diversity loss through species dissimilarity. Such effects have recently been also demonstrated for fragmented habitats (Gonçalves‐Souza et al., 2025), where lower *γ*‐diversity was linked to *α*‐diversity losses, despite species turnover among sites. This highlights the necessity of examining biodiversity at multiple scales rather than focusing solely on *α*‐diversity, as is most commonly done. Studies examining multiple spatial scales within the same experimental framework for bats, beetles and hoverflies have demonstrated that habitat heterogenization can increase *β*‐diversity (Massó Estaje et al., 2026; Mitesser et al., 2026; Wild et al., 2026). In some taxa like spiders but also understorey plants, nematodes and birds, the contribution of *β*‐diversity to change in *γ*‐diversity remained limited, with patterns largely linked to *α*‐diversity (Bradler et al., 2025; Schwarz et al., 2026; Wild et al., 2026). Furthermore, spiders were the only taxon in the BETA‐FOR experiment showing negative effects of forest heterogenization on local *α*‐diversity. Additionally, we must be aware that under the multiplicative identity of the diversity decomposition performed in this analysis, a decline in *α* mechanically inflates *β* if *γ* declines less steeply than *α*, making the *β* increase partly a mathematical artefact of *α* loss rather than genuine compensatory turnover among patches. In turn, *β*‐diversity responses could be a consequence rather than a cause that might or might not compensate for *α*‐diversity changes. However, the simultaneous positive response in functional diversity supports the idea of a true species‐sorting effect driving increases in *β*‐diversity (Mitesser et al., 2026). In turn, the results of our standardized forest experiment largely mirrors the results of recent correlative meta‐analyses based on heterogeneous datasets (Gonçalves‐Souza et al., 2025; Keck et al., 2025) and underscores the need for a better mechanistic understanding of why *β*‐diversity responses differ in order to better anticipate potential biodiversity losses as a consequence of anthropogenic impacts.

While some studies report higher species richness in open habitats and semi‐open habitat patches (Košulič et al., 2016; Plath et al., 2024) compared with the closed canopies in our control districts, much of this apparent increase may reflect insufficient considerations of the sampling effort rather than genuinely higher richness. Often, these comparisons are based on raw species counts (Chao & Jost, 2012; Gotelli & Colwell, 2001) that had been treated as if they were unbiased estimates of richness. This may lead to critical misinterpretation when the sampling effort (i.e. sample coverage) is systematically affected by environmental gradients or treatments (Kortmann et al., 2025; Rothacher, Seidl, et al., 2025). In post‐disturbance spruce patches, standardizing coverage eliminated apparent richness differences found in raw data (Plath et al., 2024). In our study, ESBC patches were dominated by cursorial species, which are more likely to be captured in pitfall traps, leading to higher local abundance and higher observed coverage (Figure S11). Among treatment types within ESBC, however, sample coverage did not differ (Figure S14). Thus, the higher crown biomass (i.e. habitat amount) in control forests and their associated spider communities likely reduced pitfall trap efficiency, potentially underestimating their diversity if sampling effects are not corrected.

An earlier fogging study at one of our sites previously revealed high canopy diversity and stochastic recolonization dynamics on mature beech trees after winter losses by wind (Hsieh, 2011). These processes result in less environmentally filtered communities in the canopy than in lower vegetation (Hsieh & Linsenmair, 2011), a pattern confirmed in a large‐scale replicated comparison of gaps and mature stands (Müller et al., 2022). Given the high dispersal capacity of spiders (Müller et al., 2022), an incomplete colonization of ESBC patches at the scale of our experiment can be ruled out. Meta‐community analyses indicate that ballooning (aerial drifting using silk) enables broad dispersal, allowing species to respond quickly to local disturbances. Species replacement was driven primarily by dispersal and habitat filtering, consistent with findings from other studies reshaping local communities (Carvalho et al., 2020; Plath et al., 2021; Samu et al., 2021). Consequently, spider diversity was lower on ESBC patches because remnant forest species inhabiting canopies and tree trunks, such as cobweb spiders (Theridiidae) like *Neottiura bimaculata*, were reduced and insufficiently replaced by settlers benefiting from increased light, structural diversity and variable humidity.

### 4.2 Functional and phylogenetic diversity responses indicate ecological similarity

We consistently observed the strongest effects of ESBC on taxonomic spider diversity, followed by phylogenetic and functional responses. This hierarchy suggests high ecological similarity, where multiple spider species perform redundant roles. Functional similarity, or niche overlap, can act as an insurance mechanism against environmental fluctuations, buffering communities against species loss so that ecosystem process rates remain stable (Eisenhauer et al., 2023). While little is known about the rates of species versus trait loss and turnover in invertebrates, functional loss and decreased niche overlap in spiders have been linked to anthropogenic disturbance (Potapov et al., 2020), as well as canopy homogenization (Wildermuth et al., 2023). Conversely, silvicultural interventions in the form of ESBC treatments acted as a habitat filter and reduced the functional diversity of spiders in the whole district. Similar filtering effects have been shown for spiders in canopy gaps of different sizes (Müller et al., 2022).

### 4.3 Limitations to sampling spiders with pitfall traps

Because spiders utilize three‐dimensional forest space, pitfall traps alone can be biased towards large, mobile ground‐dwellers (Turnbull, 1973). Nevertheless, more than half of the community in the full BETA‐FOR spider dataset obtained with pitfall traps are from the vegetation strata (Figure S16c). In line with previous pitfall studies (Müller et al., 2022), we were able to capture a number of tree‐dwelling spider species like *Tetragnatha pinicola* (Tetragnathidae) or *Diaea dorsata* (Thomisidae) which thrive on leaves and branches, as well as camouflaged trunk‐hunters like *Clubiona caerulescens* (Clubionidae) with pitfall traps in stands with a closed canopy, likely due to their seasonal descent for mating during our sampling period. Thus, the large responses of rare (*q* = 0) spiders in pitfall traps of our dataset are primarily driven by these species and their functional and phylogenetic traits. On open‐canopy patches, also in line with Müller et al. (2022), we were not able to capture these canopy‐dwelling species (Figure 2). This suggests that they either move shorter distances when on the ground or simply occur at lower densities in areas where trees are sparse.

Furthermore, a comparison of species collected with pitfall traps, Malaise traps and canopy fogging from one of our regions demonstrates that pitfall traps collect by far the most species of the regional forest species pool and represent a functionally diverse community. Nevertheless, as any method comes with bias, we encourage future studies to employ targeted combinations of methods across forest strata, including canopy fogging (Hsieh & Linsenmair, 2011; Müller et al., 2018), cross‐window traps (Niedobová et al., 2025) and vegetation sampling using Malaise traps to complement pitfall trapping.

## 5 CONCLUSION

Conceptually, our experiment refines classic ideas about habitat heterogeneity and meta‐community dynamics. Structural heterogenization increased *β*‐diversity, as predicted by the habitat heterogeneity hypothesis, but because it operated primarily through habitat filtering, this turnover coincided with, and did not compensate for, losses of *α*‐ and *γ*‐diversity. By decomposing taxonomic, functional and phylogenetic diversity along Hill numbers and across *α*, *β* and *γ* components, our results illustrate that higher *β*‐diversity cannot be interpreted as a proxy for higher regional spider diversity, and that vertical forest structure represents a key, scale‐dependent axis of niche space for arboreal predators. In this sense, the study highlights how the interplay between habitat filtering and niche overlap within meta‐communities determines whether environmental heterogeneity translates into net biodiversity gains or losses.

## AUTHOR CONTRIBUTIONS

Jörg Müller conceived the ideas and designed the methodology. Julia Rothacher, Clara Wild, Michael Junginger and Lisa Köstler‐Albert collected the data. Jean‐Léonard Stör identified the spiders. Julia Rothacher and Jean‐Léonard Stör analysed the data. Anne Chao and Oliver Mitesser provided resources for the analysis. Julia Rothacher visualized the results. Julia Rothacher and Jean‐Léonard Stör led the writing of the manuscript. Marc W. Cadotte, Akira S. Mori and Maike Huszarik supported the writing of the manuscript with their comments. All authors gave final approval for publication.

## CONFLICT OF INTEREST STATEMENT

The authors declare that they have no known competing financial interests or personal relationships that could have appeared to influence the work reported in this paper.

## STATEMENT ON INCLUSION

Our study brings together authors from Germany, Japan, Canada and Taiwan, with Germany being the country where the study was carried out. All authors were engaged with the research and study design to ensure that their diverse sets of perspectives were considered. Whenever relevant, literature published by regional scientists was cited.

## Supporting information


**Figure S1.** Schematic illustration of an experimental forest site of the ΒETA‐FOR research unit. One site consists of two different districts with the same number of patches. In the ESBC (Enhancing Structural Beta Complexity) district, manipulations were performed to increase forest heterogeneity by creating canopy gaps and deadwood structures. The control district consisted of homogeneous, untreated patches with a closed canopy. The manipulations in the ESBC district were either performed spatially aggregated in the patch center, creating a central canopy gap, or distributed throughout the patch to maintain the closed canopy. The manipulations either removed the manipulated trees completely or created different types of deadwood structures: stumps, crowns, snags, logs, snags + logs and habitat trees. Grey‐coloured treatments were applied only in the University Forest region. Two pitfall traps were installed at the central area of each patch to assess spider diversity.
**Figure S2.** Results of meta‐analysis at the patch‐scale *α*‐diversity for *q* = 0. Blue squares represent predicted means for each of the 11 sites, the blue diamond in the bottom row shows the result of the overall meta‐analysis; error bars denote 95% confidence intervals at a fixed coverage level of 0.76. W(fixed) shows the weighted effect of each site to the meta‐analysis in percent. LCL and UCL indicate the mean difference results for the lowest (LCL) and highest (UCL) estimated coverage level. Standardized diversity differences are calculated between each pair of control and ESBC districts for taxonomic (TD) and phylogenetic (PD) and functional diversity (FD). Positive values indicate an increase in diversity from control to structurally enhanced districts, negative values indicate a decrease in diversity.
**Figure S3.** Results of meta‐analysis at the patch‐scale *α*‐diversity for *q* = 1. Blue squares represent predicted means for each of the 11 sites, the blue diamond in the bottom row shows the result of the overall meta‐analysis; error bars denote 95% confidence intervals at a fixed coverage level of 0.76. W(fixed) shows the weighted effect of each site to the meta‐analysis in percent. LCL and UCL indicate the mean difference results for the lowest (LCL) and highest (UCL) estimated coverage level. Standardized diversity differences are calculated between each pair of control and ESBC districts for taxonomic (TD) and phylogenetic (PD) and functional diversity (FD). Positive values indicate an increase in diversity from control to structurally enhanced districts, negative values indicate a decrease in diversity.
**Figure S4.** Results of meta‐analysis at the patch‐scale *α*‐diversity for *q* = 2. Blue squares represent predicted means for each of the 11 sites, the blue diamond in the bottom row shows the result of the overall meta‐analysis; error bars denote 95% confidence intervals at a fixed coverage level of 0.76. W(fixed) shows the weighted effect of each site to the meta‐analysis in percent. LCL and UCL indicate the mean difference results for the lowest (LCL) and highest (UCL) estimated coverage level. Standardized diversity differences are calculated between each pair of control and ESBC districts for taxonomic (TD) and phylogenetic (PD) and functional diversity (FD). Positive values indicate an increase in diversity from control to structurally enhanced districts, negative values indicate a decrease in diversity.
**Figure S5.** Results of meta‐analysis for multiplicative (1 − S) *β*‐diversity for *q* = 0. Blue squares represent predicted means for each of the 11 sites, the blue diamond in the bottom row shows the result of the overall meta‐analysis; error bars denote 95% confidence intervals at a fixed coverage level of 0.76. W(fixed) shows the weighted effect of each site to the meta‐analysis in percent. LCL and UCL indicate the mean difference results for the lowest (LCL) and highest (UCL) estimated coverage level. Standardized diversity differences are calculated between each pair of control and ESBC districts for taxonomic (TD) and phylogenetic (PD) and functional diversity (FD). Positive values indicate an increase in diversity from control to structurally enhanced districts, negative values indicate a decrease in diversity.
**Figure S6.** Results of meta‐analysis for multiplicative (1 − S) *β*‐diversity for *q* = 1. Blue squares represent predicted means for each of the 11 sites, the blue diamond in the bottom row shows the result of the overall meta‐analysis; error bars denote 95% confidence intervals at a fixed coverage level of 0.76. W(fixed) shows the weighted effect of each site to the meta‐analysis in percent. LCL and UCL indicate the mean difference results for the lowest (LCL) and highest (UCL) estimated coverage level. Standardized diversity differences are calculated between each pair of control and ESBC districts for taxonomic (TD) and phylogenetic (PD) and functional diversity (FD). Positive values indicate an increase in diversity from control to structurally enhanced districts, negative values indicate a decrease in diversity.
**Figure S7.** Results of meta‐analysis for multiplicative (1 − S) *β*‐diversity for *q* = 2. Blue squares represent predicted means for each of the 11 sites, the blue diamond in the bottom row shows the result of the overall meta‐analysis; error bars denote 95% confidence intervals at a fixed coverage level of 0.76. W(fixed) shows the weighted effect of each site to the meta‐analysis in percent. LCL and UCL indicate the mean difference results for the lowest (LCL) and highest (UCL) estimated coverage level. Standardized diversity differences are calculated between each pair of control and ESBC districts for taxonomic (TD) and phylogenetic (PD) and functional diversity (FD). Positive values indicate an increase in diversity from control to structurally enhanced districts, negative values indicate a decrease in diversity.
**Figure S8.** Results of meta‐analysis for landscape‐scale *γ*‐diversity for q = 0. Blue squares represent predicted means for each of the 11 sites, the blue diamond in the bottom row shows the result of the overall meta‐analysis; error bars denote 95% confidence intervals at a fixed coverage level of 0.76. W(fixed) shows the weighted effect of each site to the meta‐analysis in percent. LCL and UCL indicate the mean difference results for the lowest (LCL) and highest (UCL) estimated coverage level. Standardized diversity differences are calculated between each pair of control and ESBC districts for taxonomic (TD) and phylogenetic (PD) and functional diversity (FD). Positive values indicate an increase in diversity from control to structurally enhanced districts, negative values indicate a decrease in diversity.
**Figure S9.** Results of meta‐analysis for landscape‐scale *γ*‐diversity for *q* = 1. Blue squares represent predicted means for each of the 11 sites, the blue diamond in the bottom row shows the result of the overall meta‐analysis; error bars denote 95% confidence intervals at a fixed coverage level of 0.76. W(fixed) shows the weighted effect of each site to the meta‐analysis in percent. LCL and UCL indicate the mean difference results for the lowest (LCL) and highest (UCL) estimated coverage level. Standardized diversity differences are calculated between each pair of control and ESBC districts for taxonomic (TD) and phylogenetic (PD) and functional diversity (FD). Positive values indicate an increase in diversity from control to structurally enhanced districts, negative values indicate a decrease in diversity.
**Figure S10.** Results of meta‐analysis for landscape‐scale *γ*‐diversity for *q* = 2. Blue squares represent predicted means for each of the 11 sites, the blue diamond in the bottom row shows the result of the overall meta‐analysis; error bars denote 95% confidence intervals at a fixed coverage level of 0.76. W(fixed) shows the weighted effect of each site to the meta‐analysis in percent. LCL and UCL indicate the mean difference results for the lowest (LCL) and highest (UCL) estimated coverage level. Standardized diversity differences are calculated between each pair of control and ESBC districts for taxonomic (TD) and phylogenetic (PD) and functional diversity (FD). Positive values indicate an increase in diversity from control to structurally enhanced districts, negative values indicate a decrease in diversity.
**Figure S11.** Observed sample coverage of spider pitfall samples per patch (alpha level). Sample coverage (SC) was higher on ESBC patches (treatment E) versus control patches (treatment C), indicating a more complete sampling of spider diversity in heterogeneous, more open forests with pitfall traps. Significant differences (Significance level: *p < 0.05) in observed sample coverage across treatments according to a generalized additive model with SC as a response and treatment (C vs. E). The model was controlled for the geographic location of the traps (see R‐script 07 for details).
**Figure S12.** Optimizing SES MFPD (null model using label shuffling) along an increasing a‐value, which gives an increasing weight to phylogenetic distance PDist compared with functional distance FDist from 0 meaning only functional distances are used to 100% only phylogenetic distances are used. The difference in MFPD between communities that differ in their FDist‐PDist covariances by an amount ∆COV given by the following formula adapted from Cadotte et al. (2013).
**Figure S13.** Meta‐analysis of the spider diversity response across 11 experimental forest sites at a fixed coverage level of 0.76 for a reduced dataset without the additional treatments in U01‐U03 (see Figure S1, grey). To test robustness, we removed from U01‐U03 both the six unique treatment types (tree removal, crowns and habitat trees) as well as six patches from the control districts and reran the meta‐analysis. Results are shown for landscape‐scale *γ*‐diversity (a), patch‐scale *α*‐diversity (b) and multiplicative (1 − S) *β*‐diversity (c). Points represent predicted means; error bars denote 95% confidence intervals. Standardized diversity differences are calculated between each pair of control and treatment districts in directly comparable species‐equivalent units for *γ*‐ and *α*‐diversity, and for taxonomic (TD, blue), phylogenetic (PD, green) and functional diversity (FD, yellow) across three diversity orders (*q* = 0, 1, 2). Positive values indicate an increase in diversity from control to structurally enhanced districts, negative values indicate a decrease in diversity. Effects are significant effects when the confidence intervals do not include zero.
**Figure S14.** Effects of treatment type on estimated taxonomic diversity (TD) within the ESBC district derived from generalized additive models. Note that additional treatment types only present in the University forest are excluded from this analysis due to insufficient replication for diversity estimation on an alpha level. TD was calculated at a fixed sample coverage of 0.9 along Hill numbers (*q* = 0, 1, 2). The effects of the different treatments are given relative to the treatment type with no interventions (i.e. control) in each ESBC district (closed canopy, no deadwood enrichment). Multiplicative effects from generalized additive models were controlled for the geographic location of the traps (For details on methodology see R‐Script 07). Significance codes in the bars refer to *p*‐values from the generalized additive models: * *p* < 0.05.
**Figure S15.** Venn Diagram of observed spider species in the University Forest, Sailershausen, Germany, comparing three sampling methods aimed at different strata. In May, June and July 2022, 171 spider species were caught using pitfall traps as used in the main manuscript, aimed at catching predominantly ground‐active spiders. In the same sampling interval and region, 90 species were identified through OTUs from Malaise traps, catching predominantly spiders inhabiting the herb and shrub layer. We additionally took fogging data of a published study comprising 78 species caught in May, June and July 2011 (Hsieh & Linsenmair, 2011) for comparison, as their results showed a conclusive image of the canopy and trunk dwelling spiders in the University Forest. Our Venn Diagram showed that pitfall traps can catch a large proportion of those species inhabiting higher strata, and, while it is clear that there are always specialist species that may be missed using either method, pitfall traps caught by far the largest proportion of spider species inhabiting the University forest, thus proving to be the best method to assess spider diversity if used independently.
**Figure S16.** Piecharts of percentual inhabited strata by (a) all spider species from the University Forest, Sailershausen, caught with three different strata‐appropriate methods as in S15, (b) only the species caught by pitfall trap in the University Forest and (c) all species caught by pitfall trap on all 11 BETA‐FOR sites. Spiders were ecologically classified, similarly to the traits dataset for FD, into six distinct inhabited forest strata (below‐ground layer, leaf litter layer, herb layer, shrub layer, trunk layer and canopy layer), of which a species could inhabit one or several layers.

## Data Availability

Data and code are publicly available on Zenodo as Stör & Rothacher (2026) https://doi.org/10.1101/2025.08.29.673149.
