# Supplementary material for: Temperate forest heterogeneity decreases local and landscape‐scale spider diversity through habitat filtering despite increasing species turnover
Source: J Anim Ecol. 2026 Jun 26;95(9):1793–806. doi: 10.1111/1365-2656.70297 (PMC13536275; doi:10.1111/1365-2656.70297)
Supplement: Supplementary file 1 [file JANE-95-1793-s001.docx]

**Supplementary Material**

**Figure S1** Schematic illustration of an experimental forest site of the ΒETA-FOR research unit. One site consists of two different districts with the same number of patches. In the ESBC (Enhancing Structural Βeta Complexity) district, manipulations were performed to increase forest heterogeneity by creating canopy gaps and deadwood structures. The control district consisted of homogeneous, untreated patches with a closed canopy. The manipulations in the ESBC district were either performed spatially aggregated in the patch center, creating a central canopy gap, or distributed throughout the patch to maintain the closed canopy. The manipulations either removed the manipulated trees completely or created different types of deadwood structures: stumps, crowns, snags, logs, snags + logs, and habitat trees. Grey-colored treatments were applied only in the University Forest region. Two pitfall traps were installed at the central area of each patch to assess spider diversity.

**Figure S2** Results of metaanalysis at the patch-scale α-diversity for q=0. Blue squares represent predicted means for each of the eleven sites, the blue diamond in the bottom row shows the result of the overall metaanalysis; error bars denote 95% confidence intervals at a fixed coverage level of 0.76. W(fixed) shows the weighted effect of each site to the metaanalysis in percent. LCL and UCL indicate the mean difference results for the lowest (LCL) and highest (UCL) estimated coverage level. Standardized diversity differences are calculated between each pair of control and ESBC districts for taxonomic (TD) and phylogenetic (PD) and functional diversity (FD). Positive values indicate an increase in diversity from control to structurally enhanced districts, negative values indicate a decrease in diversity.

**Figure S3** Results of metaanalysis at the patch-scale α-diversity for q=1. Blue squares represent predicted means for each of the eleven sites, the blue diamond in the bottom row shows the result of the overall metaanalysis; error bars denote 95% confidence intervals at a fixed coverage level of 0.76. W(fixed) shows the weighted effect of each site to the metaanalysis in percent. LCL and UCL indicate the mean difference results for the lowest (LCL) and highest (UCL) estimated coverage level. Standardized diversity differences are calculated between each pair of control and ESBC districts for taxonomic (TD) and phylogenetic (PD) and functional diversity (FD). Positive values indicate an increase in diversity from control to structurally enhanced districts, negative values indicate a decrease in diversity.

**Figure S4** Results of metaanalysis at the patch-scale α-diversity for q=2. Blue squares represent predicted means for each of the eleven sites, the blue diamond in the bottom row shows the result of the overall metaanalysis; error bars denote 95% confidence intervals at a fixed coverage level of 0.76. W(fixed) shows the weighted effect of each site to the metaanalysis in percent. LCL and UCL indicate the mean difference results for the lowest (LCL) and highest (UCL) estimated coverage level. Standardized diversity differences are calculated between each pair of control and ESBC districts for taxonomic (TD) and phylogenetic (PD) and functional diversity (FD). Positive values indicate an increase in diversity from control to structurally enhanced districts, negative values indicate a decrease in diversity.

**Figure S5** Results of metaanalysis for multiplicative (1 − S) β-diversity for q=0. Blue squares represent predicted means for each of the eleven sites, the blue diamond in the bottom row shows the result of the overall metaanalysis; error bars denote 95% confidence intervals at a fixed coverage level of 0.76. W(fixed) shows the weighted effect of each site to the metaanalysis in percent. LCL and UCL indicate the mean difference results for the lowest (LCL) and highest (UCL) estimated coverage level. Standardized diversity differences are calculated between each pair of control and ESBC districts for taxonomic (TD) and phylogenetic (PD) and functional diversity (FD). Positive values indicate an increase in diversity from control to structurally enhanced districts, negative values indicate a decrease in diversity.

**Figure S6** Results of metaanalysis for multiplicative (1 − S) β-diversity for q=1. Blue squares represent predicted means for each of the eleven sites, the blue diamond in the bottom row shows the result of the overall metaanalysis; error bars denote 95% confidence intervals at a fixed coverage level of 0.76. W(fixed) shows the weighted effect of each site to the metaanalysis in percent. LCL and UCL indicate the mean difference results for the lowest (LCL) and highest (UCL) estimated coverage level. Standardized diversity differences are calculated between each pair of control and ESBC districts for taxonomic (TD) and phylogenetic (PD) and functional diversity (FD). Positive values indicate an increase in diversity from control to structurally enhanced districts, negative values indicate a decrease in diversity.

**Figure S7** Results of metaanalysis for multiplicative (1 − S) β-diversity for q=2. Blue squares represent predicted means for each of the eleven sites, the blue diamond in the bottom row shows the result of the overall metaanalysis; error bars denote 95% confidence intervals at a fixed coverage level of 0.76. W(fixed) shows the weighted effect of each site to the metaanalysis in percent. LCL and UCL indicate the mean difference results for the lowest (LCL) and highest (UCL) estimated coverage level. Standardized diversity differences are calculated between each pair of control and ESBC districts for taxonomic (TD) and phylogenetic (PD) and functional diversity (FD). Positive values indicate an increase in diversity from control to structurally enhanced districts, negative values indicate a decrease in diversity.

**Figure S8** Results of metaanalysis for landscape-scale γ-diversity for q=0. Blue squares represent predicted means for each of the eleven sites, the blue diamond in the bottom row shows the result of the overall metaanalysis; error bars denote 95% confidence intervals at a fixed coverage level of 0.76. W(fixed) shows the weighted effect of each site to the metaanalysis in percent. LCL and UCL indicate the mean difference results for the lowest (LCL) and highest (UCL) estimated coverage level. Standardized diversity differences are calculated between each pair of control and ESBC districts for taxonomic (TD) and phylogenetic (PD) and functional diversity (FD). Positive values indicate an increase in diversity from control to structurally enhanced districts, negative values indicate a decrease in diversity.

**Figure S9** Results of metaanalysis for landscape-scale γ-diversity for q=1. Blue squares represent predicted means for each of the eleven sites, the blue diamond in the bottom row shows the result of the overall metaanalysis; error bars denote 95% confidence intervals at a fixed coverage level of 0.76. W(fixed) shows the weighted effect of each site to the metaanalysis in percent. LCL and UCL indicate the mean difference results for the lowest (LCL) and highest (UCL) estimated coverage level. Standardized diversity differences are calculated between each pair of control and ESBC districts for taxonomic (TD) and phylogenetic (PD) and functional diversity (FD). Positive values indicate an increase in diversity from control to structurally enhanced districts, negative values indicate a decrease in diversity.

**Figure S10** Results of metaanalysis for landscape-scale γ-diversity for q=2. Blue squares represent predicted means for each of the eleven sites, the blue diamond in the bottom row shows the result of the overall metaanalysis; error bars denote 95% confidence intervals at a fixed coverage level of 0.76. W(fixed) shows the weighted effect of each site to the metaanalysis in percent. LCL and UCL indicate the mean difference results for the lowest (LCL) and highest (UCL) estimated coverage level. Standardized diversity differences are calculated between each pair of control and ESBC districts for taxonomic (TD) and phylogenetic (PD) and functional diversity (FD). Positive values indicate an increase in diversity from control to structurally enhanced districts, negative values indicate a decrease in diversity.

**Figure S11** Observed sample coverage of spider pitfall samples per patch (alpha-level). Sample coverage (SC) was higher on ESBC patches (treatment E) vs. control patches (treatment C), indicating a more complete sampling of spider diversity in heterogeneous, more open forests with pitfall traps. Significant differences (Significance level: *p < 0.05) in observed sample coverage across treatments according to a generalized additive model with SC as a response and treatment (C vs E). The model was controlled for the geographic location of the traps (see R script 07 for details)

**Figure S12** Optimizing SES MFPD (nullmodel using label shuffling) along an increasing a-value, which gives an increasing weight to phylogenetic distance PDist compared to functional distance FDist from 0 meaning only functional distances are used to 100% only phylogenetic distances are used. The difference in MFPD between communities that differ in their FDist-PDist covariances by an amount ∆COV given by the following formula adapted from Cadotte et al. (2013):

$$\Delta MFPD= -a(1-a)\left[ \frac{(MFD)(MPD)}{{({aMPD}^{2}+\left( {1-a)MFD}^{2} \right)}^{\frac{3}{2}}} \right]\Delta COV$$

Based on the highest R² at 10% phylogeny the final functional diversity was calculated.

**Figure S13** Meta-analysis of the spider diversity response across 11 experimental forest sites at a fixed coverage level of 0.76 for a reduced dataset without the additional treatments in U01-U03 (see Fig S1, grey). To test robustness, we removed from U01-U03 both the six unique treatment types (tree removal, crowns and habitat trees) as well as six patches from the control districts and reran the meta-analysis. Results are shown for landscape-scale γ-diversity (a), patch-scale α-diversity (b) and multiplicative (1 − S) β-diversity (c). Points represent predicted means; error bars denote 95% confidence intervals. Standardized diversity differences are calculated between each pair of control and treatment districts in directly comparable species-equivalent units for γ- and α-diversity, and for taxonomic (TD, blue), phylogenetic (PD, green), and functional diversity (FD, yellow) across three diversity orders (q = 0, 1, 2). Positive values indicate an increase in diversity from control to structurally enhanced districts, negative values indicate a decrease in diversity. Effects are significant effects when the confidence intervals do not include zero.

**Figure S14** Effects of treatment type on estimated taxonomic diversity (TD) within the ESBC district derived from generalized additive models. Note that additional treatment types only present in the University forest are excluded from this analysis due to insufficient replication for diversity estimation on an alpha level. TD was calculated at a fixed sample coverage of 0.9 along Hill numbers (*q* = 0, 1, 2). The effects of the different treatments are given relative to the treatment type with no interventions (i.e. control) in each ESBC district (closed canopy, no deadwood enrichment). Multiplicative effects from generalized additive models were controlled for the geographic location of the traps (For details on methodology see R-Script 07). Significance codes in the bars refer to *p*-values from the generalized additive models: * p < 0.05.

**Figure S15** Venn-Diagram of observed spider species in the University Forest, Sailershausen, Germany comparing three sampling methods aimed at different strata. In May, June and July 2022, 171 spider species were caught using pitfall traps as used in the main manuscript, aimed at catching predominantly ground-active spiders. In the same sampling interval and region, 90 species were identified through OTUs from Malaise traps, catching predominantly spiders inhabiting the herb and shrub layer. We additionally took fogging data of a published study comprising 78 species caught in May, June and July 2011 (Hsieh & Linsenmair, 2011) for comparison, as their results showed a conclusive image of the canopy and trunk dwelling spiders in the University Forest. Our Venn-Diagram showed that pitfall traps can catch a large proportion of those species inhabiting higher strata, and, while it is clear that there are always specialist species that may be missed using either method, pitfall traps caught by far the largest proportion of spider species inhabiting the University forest, thus proving to be the best method to assess spider diversity if used independently.

**Figure S16** Piecharts of percentual inhabited strata by a) all spider species from the University Forest, Sailershausen, caught with three different strata-appropriate methods as in S15, b) only the species caught by pitfall trap in the University Forest and c) all species caught by pitfall trap on all eleven BETA-FOR sites. Spiders were ecologically classified, similarly to the traits dataset for FD, into six distinct inhabited forest strata (belowground layer, leaflitter layer, herb layer, shrub layer, trunk layer and canopy layer), of which a species could inhabit one or several layers.

**S17 Information for expanded phylogenetic tree**

The backbone phylogeny for our analysis was estimated in Müller et al. (2022) for 506 central European spiders, predominantly forest inhabiting species based on a data matrix of published DNA sequences assembled with the R package *megaptera* (version 1.0-38) from GenBank and BOLD repositories. Four mitochondrial (*cox1*, *nad1*, 12S rRNA gene, 16S rRNA gene) and three nuclear (H3, 18S rRNA gene, 28S rRNA gene) loci were aligned and sequences were concatenated into a single matrix. Since phylogenetic information of the seven loci cannot be expected to resolve deep nodes in the phylogeny, a topological constraint on the phylogeny based on recent phylogenomic studies was imposed (Bond et al., 2014; Fernandez-Palacios & de Nikolas, 1995; Sharma et al., 2014). Additionally, we added 13 new species from our data to this tree by connecting them to their close congener relatives or sister taxa using the *bind.tip* function of the *phytools* package (Revell, 2024).

Bond, J. E., Garrison, N., Hamilton, C., Godwin, R., Hedin, M., & Agnarsson, I. (2014). Phylogenomics Resolves a Spider Backbone Phylogeny and Rejects a Prevailing Paradigm for Orb Web Evolution. *Current Biology,* 24, 1765–71.

Fernandez-Palacios, J. M., & de Nikolas, J. P. (1995). Altitudinal Pattern of Vegetation Variation on Tenerife. *Journal of Vegetation Science,* 6, 183–90.

Müller, J., Brandl, R., Cadotte, M. W., Heibl, C., Bässler, C., Weiß, I., Birkhofer, K., Thorn, S., & Seibold, S. (2022). A replicated study on the response of spider assemblages to regional and local processes. *Ecological Monographs*, *92*(3), 1–19. https://doi.org/10.1002/ecm.1511

Revell, L. J. (2024). phytools 2.0: an updated R ecosystem for phylogenetic comparative methods (and other things). *PeerJ*, *12*, e16505. <https://doi.org/10.7717/peerj.16505>

Sharma, P. P., Kaluziak, S. T.,  Pérez-Porro, A. R., González, V. L.,  Hormiga, G., Wheeler, W. C. & Giribet, G. (2014). Phylogenomic Interrogation of Arachnida Reveals Systematic Conflicts in Phylogenetic Signal. *Molecular Biology and Evolution,* 31: 2963–84.

**S18 Detailed description of meta-analysis**

To quantify landscape-scale effects of our experimental manipulation on spider γ-diversity, we conducted the meta-analysis across all 11 paired experimental districts using the *iNEXTmeta* function (Chao, 2025) for abundance data, with 99 bootstrap replications ensuring robust results. Because diversity data are typically incomplete and sample coverage of arthropod samples varies systematically with canopy treatment (Rothacher et al., 2025), standardization to a common coverage level within a unified framework is required for meaningful comparisons of diversity. Without correcting for such biases, diversity can be under- or overestimated, and in the worst case, apparent treatment effects may simply reflect coverage differences. For our analyses, we used a standardized coverage value of 0.76, which corresponds to the standard output value calculated by the *iNEXTmeta* function.

The *iNEXTmeta* function jointly incorporates sample coverage standardization and the calculation of diversity metrics along Hill numbers (q = 0, 1, 2) to consider species frequency distributions. This allows a focus on rare (q = 0), common (q = 1), and dominant (q = 2) species, lineages and functional units in the dataset. In our case, rarity partly reflects the sampling method, with rare species largely representing vegetation- and arboreal-dwelling taxa, whereas common and dominant species are mainly ground-dwelling. In this framework, TD quantifies the effective number of equally abundant species. PD quantifies the effective number of equally divergent lineages, while FD quantifies the effective number of equally distinct functional species. Since TD, PD, and FD are in the same units (species/lineage equivalents) they can be meaningfully compared (Chao et al., 2014). Ultimately, *iNEXTmeta* provides average effect sizes and bootstrap-derived confidence intervals aggregated across district pairs.

To identify the relevant spatial scale of treatment effects we decomposed γ-diversity (district level) into two hierarchical components within the meta-analytic framework using the *iNEXTmeta.beta* function(Chao, 2025; Massó Estaje et al., 2026; Mitesser et al., 2026): (1) within-patch diversity (α-component), and (2) among-patch diversity (β-component). The overall effect was considered significant when the 95% confidence interval did not include zero. For TD, γ-diversity quantifies the effective number of species in the pooled community across all patches of a district, while α-diversity represents the average effective number of species per patch. The interpretation for PD and FD follows analogously by replacing “species” with “lineages” or “functional units”. β-diversity is defined as the ratio of γ-diversity to α-diversity. In a district with nine patches, β-diversity equals 1 when all patches share identical species composition and abundances and reaches a maximum of 9 when no species are shared among patches, indicating complete turnover. Thus, β-diversity quantifies the degree of compositional differentiation among patches and is expressed as the effective number of distinct patches. To enable meaningful comparisons of β-diversity while accounting for variation in patch numbers across districts (i.e., 9 or 15 patches per district), we used the Jaccard-type turnover (1-S) transformation (Chao et al., 2019), which quantifies dissimilarity between assemblages relative to γ-diversity and is standardized by patch number, with zero indicating identical assemblages in the patches and one indicating no shared species.

**References:**

Chao, A. (2025). *CRAN repository iNEXT functions*. https://github.com/AnneChao/iNEXT.meta

Chao, A., Chiu, C., Wu, S.-H., Huang, C.-L., & Lin, Y. (2019). Comparing two classes of alpha diversities and their corresponding beta and (dis)similarity measures, with an application to the Formosan sika deer (*Cervus nippon taiouanus*) reintroduction program. *Methods in Ecology and Evolution*, *10*. https://doi.org/10.1111/2041-210x.13233

Chao, A., Chiu, C.-H., & Jost, L. (2014). Unifying Species Diversity, Phylogenetic Diversity, Functional Diversity, and Related Similarity and Differentiation Measures Through Hill Numbers. *Annual Review of Ecology, Evolution, and Systematics*, *45*, 297–324. https://doi.org/10.1146/annurev-ecolsys-120213-091540

Massó Estaje, C., Rothacher, J., Vujić, A., Miličić, M., Chao, A., Mitesser, O., Müller, J., Claßen, A., & Steffan-Dewenter, I. (2026).

Experimental enhancement of structural heterogeneity in forest landscapes promotes multidimensional hoverfly diversity. *Journal of Applied Ecology*, *63*(1). https://doi.org/10.1111/1365-2664.70252

Mitesser, O., Cadotte, M., Akira Mori, Plas, F. van der, Chao, A., Rothacher, J., Bässler, C., Bevanda, M., Biedermann, P., Bradler, P., Castaneda-Gomez, A., Decker, O., Delory, B. M., Dittrich, S., Fichtner, A., Kreis, A., Köstler-Albert, L., von Oheimb, G., Pflumm, L., … Müller, J. (2026). Old growth attributes by chain saw: how between-patch heterogeneity changes the metacommunities of beetles in temperate forests. *Ecology Letters*, *29*(3). https://doi.org/https://doi.org/10.1111/ele.70355

Rothacher, J., Seidl, R., Thom, D., Kortmann, M., Chao, A., Chiu, C.-H., Heibl, C., Hothorn, T., Mitesser, O., Mori, A. S., Morinière, J., Pierick, K., Wild, C., Wild, N., & Müller, J. (2025). The impact of tree mortality and post-disturbance management on insect diversity in temperate forests: Insights from a replicated experiment. *Journal of Applied Ecology*, *62*(8), 1878–1888. https://doi.org/https://doi.org/10.1111/1365-2664.70086
